# Dynamics of a single cavitation bubble near an oscillating boundary

**DOI:** 10.1038/s41598-024-73540-3

**Published:** 2024-09-25

**Authors:** Hemant J. Sagar, Yuxing Lin, Ould el Moctar

**Affiliations:** 1 Institute of Ship Technology, Ocean Engineering and Transport Systems (ISMT), University of Duisburg-Essen, Bismarckstr. 69, 47057 Duisburg, Germany; 2 Department of Hydro and Renewable Energy, Indian Institute of Technology (IIT), 247667 Roorkee, India

**Keywords:** Cavitation bubble, Collapse, Oscillating boundary, Mechanical engineering, Biomedical engineering

## Abstract

Cavitation and its effects are well investigated, especially single bubble cavitation and its collapse near rigid and elastic boundaries. In our current article, we investigated novel experiments of a single cavitation bubble near an oscillatory boundary. We generated the cavitation bubble by laser focusing in water. A flat glass plate was fixed to the shaft of the magnetostriction oscillator coil. We investigated the dynamics of bubbles at two relative wall distances (ratio of the distance between the bubble center and plate surface to the maximum radius of the bubble) of the bubble from the glass plate in combination with four modes of oscillation. Each mode has specific frequency and amplitude of oscillation. The high-speed camera captured the dynamics of the bubble using the back-illumination method with a framing rate of 120Kfps and simultaneously we used an optical CMOS sensor to measure the oscillation of the glass plate. We presented a clear comparison among the bubble dynamics near stationary and oscillating plates with parameters such as oscillating modes and direction. We correlated the dynamics of the bubble with the motion of the plate. In addition, we highlighted the differences including the characteristics of bubble shape and jetting that occurred during the collapse phase. The comparison of the time histories of the bubble’s equivalent size postulated that the bubble’s collapse times vary significantly in some cases compared to the bubble’s dynamics near the stationary plate. In all cases, we noticed the shortening of the bubble’s collapsing time, i.e. accelerated collapses. In our findings, we noticed a collapse times reduction of about 4–15%. Our finding signifies the importance of introducing the oscillation of the boundaries to obtain effective energy concentration over the time during the collapse. Our study also suggests that forced oscillation of boundaries is undesirable for destructive cavitation effects. The method we suggested for the manipulation of bubble dynamics holds potential for enhancing the efficiency of applications such as lithotripsy in biomedical devices, actuation and micro pumping in microfluidic devices, and effective semiconductor surface cleaning. Not but least, obtained results can be used as benchmark in future for validating numerical methods.

## Introduction

The high-speed flow of liquid around complex shapes such as propellers or impellers can drop the local pressure to saturation pressure of fluid causing a phase of liquid termed as cavitation. Cavitation is a very common issue in turbomachinery resulting in erosion and vibration of the component and nearby appendages. Excessive cavitation may also cause a reduction in the efficiency of the machinery. On the other hand, cavitation and its destructive mechanism which include jetting, shock wave, and shear rates on the target body are useful in various engineering applications such as emulsification^1^, dentistry^2^, dispersion^3^, surface cleaning^4^, etc. So far, microjet^5^, shock wave^6^, and shear rates^4^ are predicted as dominant damaging parameters. The range of microjet was predicted in the range of a few hundred meters per second and the impact range was so far variable in various investigations. Later 1980, it became prevalent to study laser-induced cavitation bubbles or spark-induced cavitation bubbles near solid and elastic boundaries, and free surfaces^7^. Research regarding a single cavitation bubble progressed significantly in understanding their dynamics at various boundary conditions, e.g. flat solid wall^8^, curved boundaries^9,10^, between parallel or oblique plates^4,11^. Our recent study^11^ of bubble dynamics between two oblique plates opened a new direction to manipulate the bubble dynamics and its direct application in microfluidic devices. However, in practice, the cavitation occurs near non-rigid boundaries, i.e. the nearby structures cannot be completely assumed as rigid. They are subject to vibration, such as hydrofoil^12^, ship propellers^13^, and tissues^14^. In this regard, particular attention was focused on investigating cavitation bubble dynamics near soft coatings on metal surfaces. These investigations aimed to repel the bubble’s collapse from the solid boundary and avoid erosion risks. Other directions of biomedical sciences also investigated the single bubble cavitation near membranes or biomaterials to observe the behavior of cavitation bubbles in laser-based surgeries. Blake and Gibson^7,15^ extensively investigated the bubble dynamics near the deformable and foam-like coatings concluding that stiffness and inertia are the factors that determine the type of collapse. Later on, Shaw et al.^16^, Turangan et al.^17^, Orthaber et al.^18^, and Sankin and Zhong^19^ investigated the bubble dynamics near thin membrane-like silicon sheets. Shaw et al.^16^ presented the visual relationship between bubble collapse and the induced deformation of the silicon membrane using interferometry. Sankin & Zhong determined that the largest deformation occurs when the relative wall distance is small enough. Brujan et al.^20,21^ investigated the cavitation bubble near hydrogel by changing the elastic modulus of the hydrogel at 5 million frames per second of imaging. They estimated about 960 ms^−1^ jetting velocity which penetrated the soft hydrogel at a lower relative wall distance between the bubble and the target surface. Turangan et al. studied the bubble dynamics near the membrane by both, experimental and numerical (using the boundary element method)^17^. The membrane curvature during bubble collapse is responsible for pressure building up and repelling of bubble^17^. Ohl et al.^22^ numerically investigate the bubble dynamics near elastic and rigid biomaterial like skin, tissue and bone; and their study revealed that bubble collapse on the harder material while bubble collapse away and splits into smaller bubble when it collapses near softer materials like skin. Similar to Turangan, Orthaber et al.^18^ and Shervani et al.^23^ in their numerical investigation concluded that stiffness is affecting parameter for type of bubble collapse and splitting of bubble occurs at lower relative wall distance in the range of 0.5–1.0^23^. Orthaber et al.^18^ mentioned membrane rupture by collapsing bubble. However, laser shall not coincide with the membrane surface minimizing damaging effects. Recently, Ma et al.^24^ in their investigation of bubble dynamics near rigid and elastic wall concluded that bubble’s oscillation times are lower for bubble dynamics near elastic boundary than the rigid boundary. They also postulated that the jetting mechanism was significantly different both cases. Same as previously reported in several investigations, Xu et al.^25^ in their experimental investigation reported that elasticity modulus of the nearby boundary and relative wall distance influences the bubble dynamics. Elastic boundary stores the energy during the bubble’s expansion and releases during collapse phases^26^. Apart from experimental and numerical investigation, there exist several coupled numerical investigations between computational fluid dynamics and finite element method^9,27–30^. The typical computational fluid method used as either boundary element method^9^ or volume of fluid method^28^ or smooth particle hydrodynamics^31^. These investigations covered material like sandwich structures^27^, polymers, nylon, Teflon plates. Few of them include hollow bodies like spheres^9^ and cylinders^32^. Most of the investigations summarized the shape of bubble, displacement of elastic structure and stress on the elastic boundary. All mentioned investigation considered the interaction between bubble and elastic structures. However, Wang et al.^33^ investigated the interaction between elastic plate, air bubble and flexible polyvinylchloride sheet. They reported jetting, splitting and coalescence of bubbles. Also Lin et al.^34^ investigated the deformation and stress on the hydrofoil subject to hydrodynamics cavitation using digital image correlation. Their findings depict that multiple frequency peaks of hydrofoil oscillation and shedding frequency of the cavitation affected the hydrofoil vibrations. In numerical and experimental investigations, Han et al.^35^ concluded the shock wave, jetting and combination of both are responsible for structural vibrations. Sieber et al.^36^ investigated the interaction between laser induced cavitation bubble and elastic material hydrogel having similar properties of biological tissues. Their numerical investigations were based on boundary integral method used by Klaseboer et al.^37^ and their experiments almost overwrite the experiments by Brujan et al.^21^ with similar material hydrogel and postulated bubble splitting and microjet. Brujan et al.^21^ predicted microjet velocity around 800 ms^−1^ while Sieber et al.^36^ predicted around 1000 ms^−1^ and atomic microjet with 2000 ms^−1^.

All mentioned investigations above are related to the bubble dynamics and its interaction with the elastic boundaries. However, in a very recent numerical study by Nguyen et al.^38^, bubble dynamics was investigated near oscillating boundary using the finite volume method. Their numerical investigation postulated that the bubble was collapsing more violently and shock wave dynamics were more dominant than the bubble’s dynamics near stationary plates. In their simulation bubble was simulated from the full-grown bubble condition. They initiated their simulation without pre-oscillating boundary, i.e. boundary started oscillating after bubble dynamics started. Therefore, the flow disturbances by the oscillating plate before the full-grown condition were not considered which might be of interest. Here, it needs to be pointed out that if boundary is already in oscillating condition, flow disturbances/flow field may vary the bubble dynamics. In their all investigations, the plate’s oscillation frequency was one-fourth of the oscillation frequency of the bubble. The amplitude varied from about one-tenth of the bubble’s maximum radius to 1.5 times the maximum bubble radius. This article was the motivation behind our work because their investigation was the missing piece of experimental results.

So far, several investigations covered extensive information about the bubble dynamics near the flat stationary plate. In their cases the boundary was non-oscillating. In general, after plasma seeding, a bubble having higher internal pressure starts to expand radially. Based on its distance from the boundary it cultivates its shape near a boundary, such as a bubble’s surface flattens when come in contact with the stationary boundary if the distance between the bubble and the stationary boundary is less than its radius. If this distance is larger than the radius then the bubble grows almost spherically without any restrictions. The bubble grows until the pressure inside the bubble and balance with the external hydrostatic pressure. Due to the speed of expansion, the bubble stretches out its expansion more therefore resulting significant lower the internal pressure of the bubble. The pressure difference results again in the compression of the bubble. In compression or collapsing phase of the bubble is considered more considerable due to its role in destruction effects. Non-uniform pressure distribution surrounding collapsing bubbles introduces the instabilities over the bubble surface, such as conical shape, notch formation, etc. The major effect of the non-spherical collapse during the first oscillation cycle is microjet impacts which were highlighted as major contributors to the cavitation damage along with shock wave impacts. In the article by Sagar and el Moctar^5^, in their experimental and numerical investigation, they clearly observed that microjet is the main damaging parameter. The microjet velocities can range up to a few hundred ms^-1^ and result from the volatile collapse of the bubble. As mentioned in the section introduction, the application asks for both advantages and disadvantages of cavitation, however in many cases, cavitation is unavoidable, such as pumps and hydro-machinery. Therefore, controlling cavitation, by either slowing it down or by increasing its speed for highly desired destructive effects is necessary.

Overall, the previous literature was missing experimental investigation of bubbles near oscillating plates. Also, for practical application or an in-depth understanding of oscillating plates and their effect on collapsing bubbles need to be carefully taken care of. Specially flow disturbance by oscillating boundary may play a role in determining cavitation bubble dynamics. Therefore, in our current work authors proposes an experimental investigation of the single bubble dynamics near oscillating rigid glass wall. We performed experiments near four different amplitudes of oscillations, where maximum bubble radius to the amplitude ratio was kept between 13–40, which was far smaller than the parameters in the investigation of Nguyen et al.^38^ (about 2-time bubble radius). We captured the bubble dynamics with a highspeed camera; and frequencies and amplitude of the oscillatory glass plate were measured using an optical displacement sensor. The set of parameters, frequency and amplitude of the oscillating glass plate is termed as *Mode (M)*. We compared results for four cases of modes of oscillations and two relative wall distances between plate and bubble. The one where bubble radius is lesser than the distance between bubble center and wall. In such case, bubble get attached (thin liquid layer exists) to the plate at its full-grown condition and later phase of its dynamics. In other case of higher relative wall distance, the bubble is generated at larger distance that its radius from the plate. Nearby boundary does not restrict the growth of bubble and clear jetting towards surface can be seen during collapse. We compared the time history of the bubble’s equivalent radius for different modes of frequencies and conclusions were made.

## Results

**Fig. 1:**
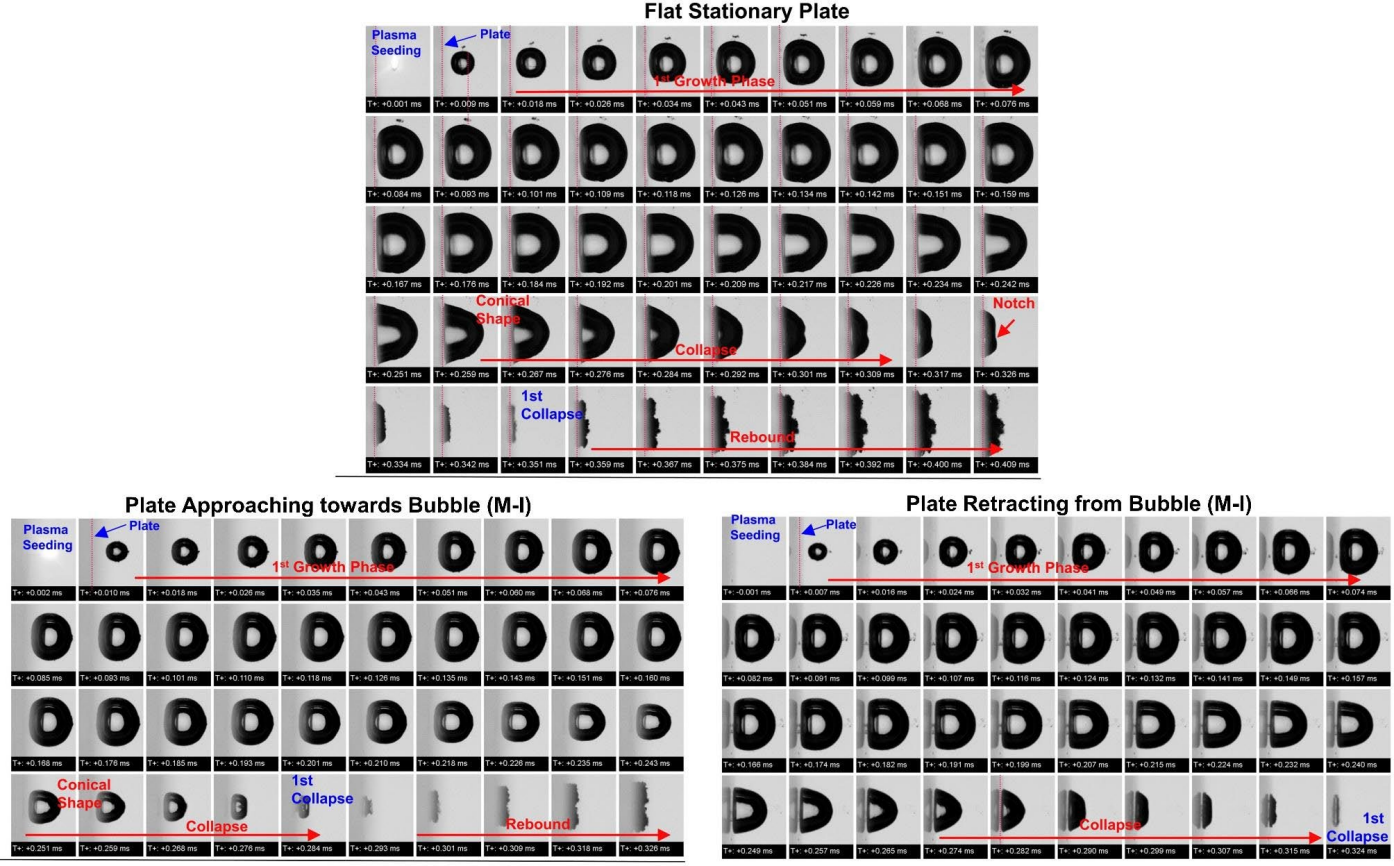
Comparison between captured imaging sequence for bubble dynamics near flat stationary plate (top), oscillating plate approaching towards bubble (bottom-left) and oscillating plate retracting from the bubble (bottom-right) for the distance between bubble center and plate surface (D) of 1 mm. Bubble dynamics have shown imaging sequence covered the first collapse starting from plasma seeding. The initial position of the plates is shown with red-dotted lines.

**Fig. 2:**
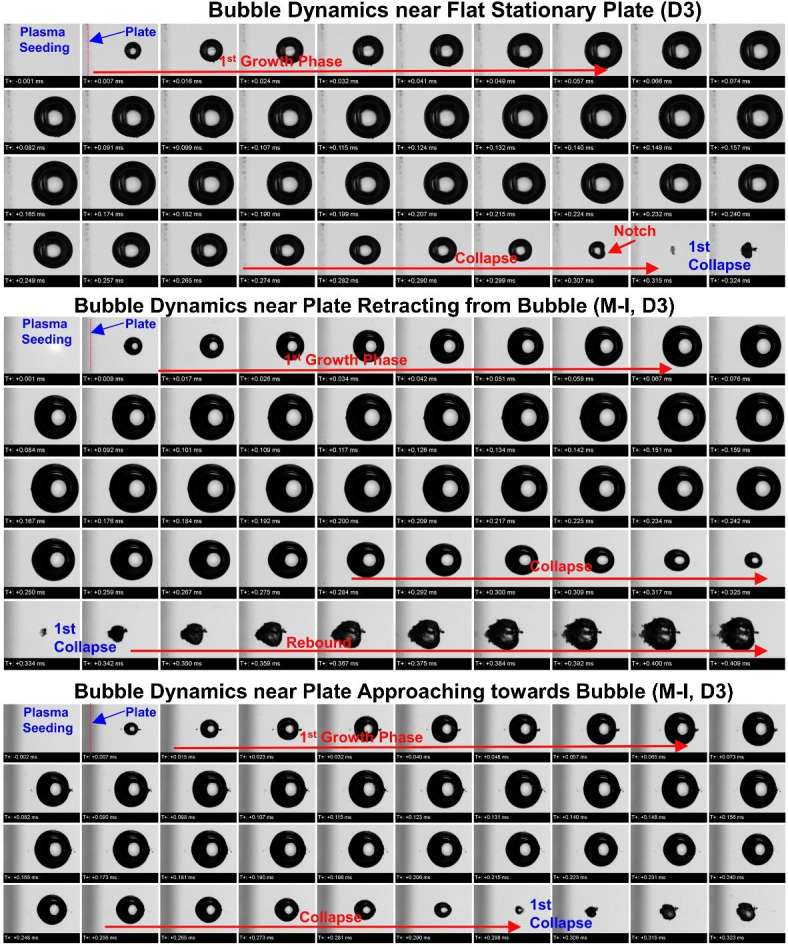
Comparison between captured imaging sequence for bubble dynamics near flat stationary plate (top), oscillating plate retracting from bubble (middle) and oscillating plate approaching towards collapsing bubble (bottom) for the distance between bubble center and plate surface (D) of 3 mm. Bubble dynamics have shown imaging sequence covered the first collapse starting from plasma seeding. The initial position of the plates is shown with red-dotted lines.

Our investigation includes a series of experiments focusing on bubble dynamics near an oscillating plate with two variable distances (annotated as D1 and D3) and four oscillation modes of a plate (annotated as M-I, M-II, M-III, and M-IV). The details of the frequency and amplitude of oscillating modes are given in Table 1. In addition, our investigation represents the two variable conditions for all considered eight test cases, the one in which the plate was moving retracting i.e. to the left from the bubble during its collapse, and in another condition when the oscillating plate was approaching towards collapsing bubble i.e. to the right side. For the representation, in the first part of the results, a comparison of bubble dynamics captured near flat and stationary plates in comparison with the bubble dynamics near plates oscillating with mode M-I are presented in Figs. 1 and 2 for two varying distances between the bubble and plate surface.

**Table 1:** Test matrix including test parameters and measurements.

Test case	Freq. modes	Amplitude	Frequency	Rmax	D	γ
	M	(mm)	(Hz)	(mm)	(mm)	–
D1	I	0.04 ± 0.003	294.784	1.35 mm ± 0.05	1 mm ± 0.04	0.74 ± 0.05
	II	0.084 ± 0.0025	270.872			
	III	0.089 ± 0.0015	333.984			
	IV	0.130 ± 0.0045	312.816			
D3	I	0.04 ± 0.003	294.784		3 mm ± 0.04	2.22 ± 0.05
	II	0.084 ± 0.0025	270.872			
	III	0.089 ± 0.0015	333.984			
	IV	0.130 ± 0.0045	312.816

From the imaging sequence in Fig. 1, one can see that the bubble collapsing in all three cases expands after plasma seeding attaining the oval shape having a flattened surface near the plate. The distance between the plate and the bubble center was less than the radius of the bubble resulting in the attachment of the bubble to the plate at its full-grown condition. The bubble’s shapes until its full-grown condition are almost identical and certainly not easy to recognize the full-grown condition. However, major discrepancies are noticed during the collapse phase among all three cases. In the case of flat plate condition, the bubble was a conical shape at the beginning of the collapse and a notch was formed before the collapse took place. The flattening was also begun about seven frames before the collapse occurred, although the bubble shrank only laterally by preserving its longitudinal stretch. In the case of the plate approaching towards bubble, the bubble remained oval shape for about two frames before it collapses and it shrank laterally and longitudinally both resulting in a compact cavity before collapse and a smaller collapsing region. In this case, notch formation was not observed before the collapse, and the bubble preserved the roundness overall periphery including places where it attached to the plate. For a case where the plate approaching towards the bubble, bubble shape showed the characteristics combined from both stationary plate and the plate retracting from bubble, such as flattened wall attached surface and conical shape. In this case, the surface attached to the wall was flattened however the thicker liquid layer was observed between the bubbles surface and the plate. Bubble during its initial collapse phase maintained its oval shape. However, it later converted to conical. The bubble shrank laterally more than longitudinally. The last stage of the bubble reflected the wide notch formation in this case, though the bubble collapsed on the plate with a wider impression than the bubble collapsing near the retracting plate from bubble and less wide than the stationary plate. Overall, the observations highlighted the significant effects on the bubble dynamics and mainly during collapsing phase. The notch formation referring to the jetting, flattening, and collapsing region are major differences in presented cases, however, one can expect varied effects raised from bubble dynamics and their applicability can be extended to desired applications.

The effect of the damage is not considered if the distance between the bubble center and the plate is double that of the bubble radius. The bubble near a flat wall for higher relative wall distance rarely shows the damaging effects of the wall, however, these cases show intense and unified characteristics of jetting. Figure 2 shows the captured imaging sequence of bubble dynamics for the distance of the plate three millimeters from the bubble center, thereby bubble got sufficient space to expand fully without attaching to the plate surface. As seen in all presented cases, the bubble grows almost spherically after plasma seeding and maintains its sphericity for most of its collapse duration. In this case, major changes are noticed about 2–4 frames before the collapse took place. In the case of a bubble collapsing near a flat stationary plate, the bubble shows a notch before the collapse and the collapse as a small concentric entity. Later regrown cavity looks like a cavity with a counter jet (tail) at its one surface and another elongated end. However, the bubble was spherical in shape until a notch formed in the surface, which subsequently collapse showing a tiny cavity. The shape of the bubble before collapse became significantly oval and stretched laterally in case of the plate retracting from bubble. Similar to the previous near plate bubble condition, in this case also notch formation was not observed. A counter jet representing the jetting during collapse was also absent in rebounded cavity. However, one can postulate that the retracting plate stretched out the collapsing bubble in elongating it to some extent and it was pronounced at the end of the collapse. The bubble dynamics near the plate approaching towards bubble, such stretching and elongation of the bubble was not observed and the bubble remained spherical until it collapses except few local instabilities. The right-side surface of the bubble is seen blurred in the collapse image thereby indicating the acceleration of the respective surface. It might have happened that the bubble did not jet out. This postulation is proved by the shape of regrowing bubble where the bubble was regrowing with a tiny counter jet but without a sharpened left surface of the bubble, which was mainly observed in previous both jetting cases. The shape of the bubble during collapse is also without a notch and seems that it collapses spherically at a tiny region. Overall, in this case, the major differences were noticed in the shape of the bubble before collapse and elongation of the left side bubble surface. The regrown shape of the bubble also indicated the presence of the jetting phenomenon during collapse by reflecting the counter jet. Somehow, we are convinced that the oscillating plate affects the dynamics of the bubble in the collapse phase.

**Fig. 3:**
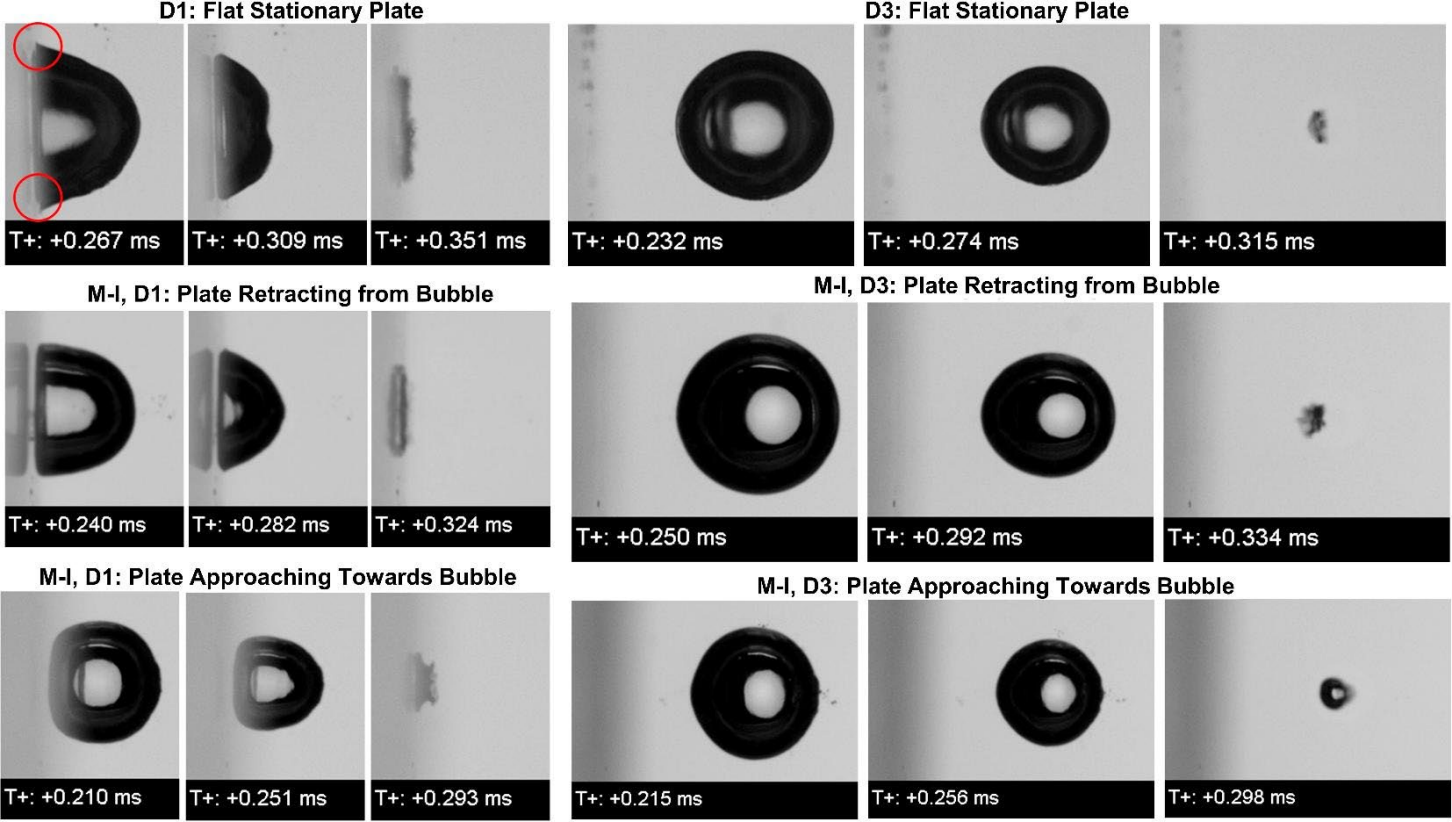
Comparison of the instances before the collapse phase showing the change in the shape and their differences based on a distance between the bubble center and the surface of the plate of D1 = 1 mm and D3 = 3 mm for the plate’s oscillating mode M-I. The red circles showed sharp corners of the bubble near plate for stationary plate.

Figures 1 and 2 elaborated more about the global differences observed regarding bubble dynamics near oscillating boundaries for two different distances between the bubble and plate. However, the comparison shown in Fig. 3 of shapes observed during the collapse phase are relevant and highlights the differences in various test condition. The notch formation took place at the surface of the bubble on the right. The notch formation is considered as the beginning of the microjet initiation and it is important for investigating the loads by jetting. Due to restricted flow from the bottom of the bubble, the pressure gradient exists on both surfaces of the bubble, the one near the plate and the opposite one. This pressure gradient pronounces when the bubble starts to violently collapse due to pressure difference. The localized higher pressure at the surface of the bubble produced the notch and triggered the jetting during the collapse. In observed cases, for D1 in Fig. 3, the notching phenomenon is only observed for bubble collapsing near the stationary plate and it was absent in cases of plate oscillating irrespective of its direction of motion. However, the differences in shapes are observed like a conical shape for the stationary plate, a conical but rounded shape during the retracting plate, and an almost rounded bubble with a flattened plate attached bubble for the plate approaching towards bubble. These differences indicate that the above-mentioned effect of pressure gradient varied significantly. Such as restricted flow and lower pressure on the plate-attached surface were compensated by plate approaching towards collapsing bubble. While the restricted the flow was made free by the plate moving away from the bubble. In both moving plate cases, the pressure on the surface was somehow by either the motion plate toward the bubble or the plate moving away from the bubble making space for flow; but certainly, lowering the pressure gradient by obscuring the possibility to distinctly jet on the surface. In addition, the major difference can be seen in the shape of the bubble at collapse, such as flattened thin toroid, less wide toroid, and thicker but concentric toroid shaped for stationary, retracting and approaching plates to the bubble, respectively. To the local extent, we observed that the edges of the bubble shown in red circles in Fig. 3 for the bubble near the plate, show the bubble’s attachment to the plate, such as the surface if the bubble attached to the plate has sharp corners, therefore, the contact angle between bubble and plate seems very small, while for rest two cases of a bubble near moving plate, the same corners of bubble appeared to be rounded and smooth. In general, our results and observation clearly augmented that notch formation thereby manipulation of jetting is possible. The direction plate can certainly decide the possible cavitation affecting spatial regime. Bubble dynamics can be simply manipulated by just oscillating the nearby boundary possibly obtaining the desired effect or avoiding the cavitation-induced destructive effects.

## Discussion

**Fig. 4:**
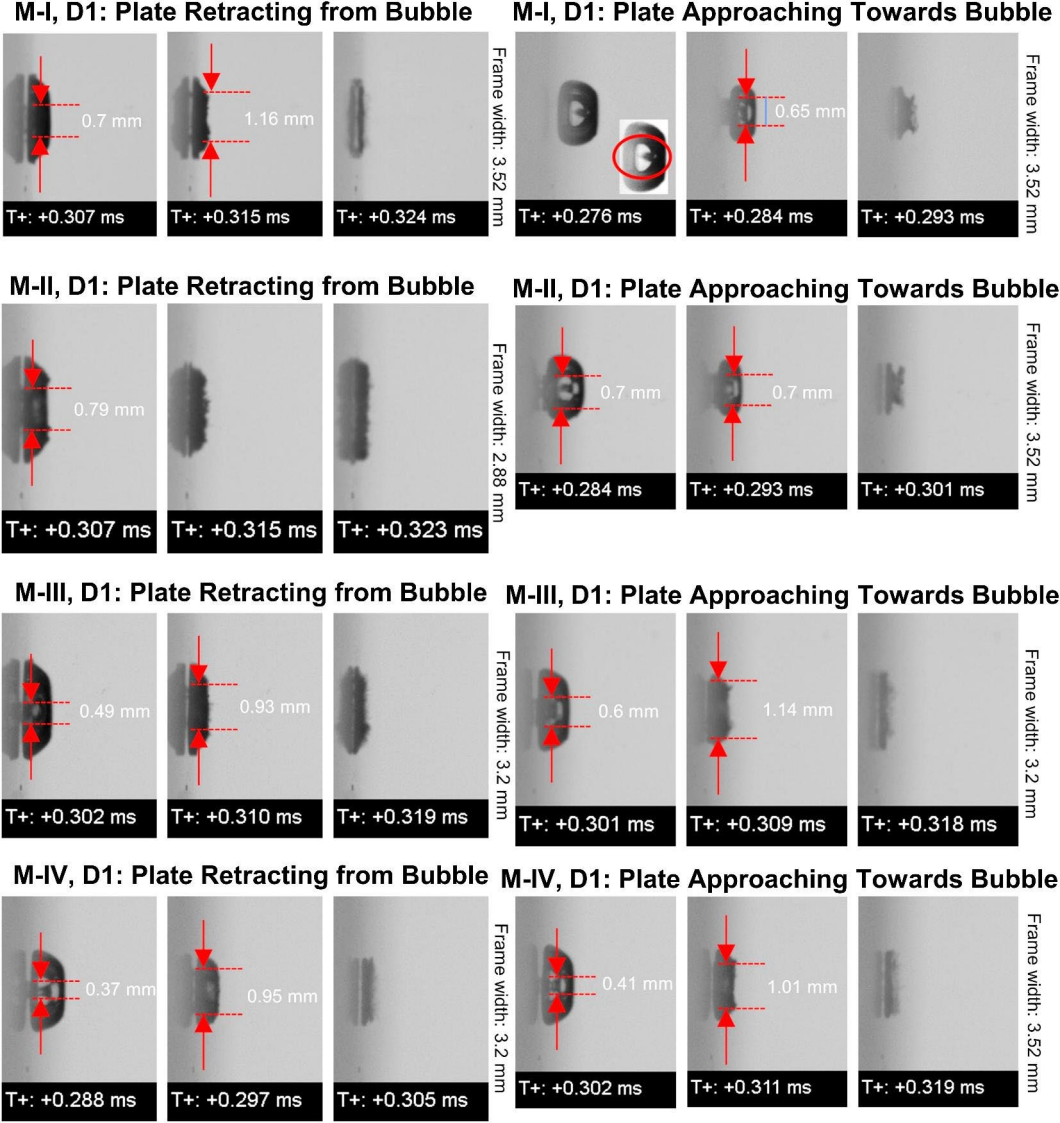
Comparison among the bubble shapes observed during four various modes of oscillation of plate in combination with the direction of plate motion for the distance (D1) between bubble center and plate equal to 1 mm. The red arrows indicate the approximate width of the jet impacting on the plate.

The previous section clearly demonstrated the significance of our proposed methodology and possible outcomes. Further, to gain more clarity on the relation between the oscillatory motion of the boundary and the bubble dynamics, four modes (see Table 1) of the oscillatory motions were investigated. In the scope of this article, we are presenting an imaging sequence representing the bubble dynamics to highlight the global differences and collapsing times for quantifying the results. Figure 4 represented the summary of the imaging sequences observed at the end of the collapse phase for both distances D1 and D3 in combination with the four oscillatory modes of the plate. The time difference of 8–9 microseconds between imaging frames are larger against the speed of bubble dynamics. However, here we relied on global bubble shape comparison and local shape uniformities not in scope of this article. We believe that the shape comparison presented may differ slightly with those would have been captured ultrahigh speed imaging.

The major difference noticed among all is the spread of the bubble’s collapsing region, which was distinct and wider in the case of the plate retracting from bubble. On the other hand, for modes I and II, and plate approaching towards bubble reflected the less wide and more rounded cavities attached to the plate. Except for these two cases, in all cases, the collapsing bubble was wider and more distinct, thicker for the plate moving to the left.

**Fig. 5:**
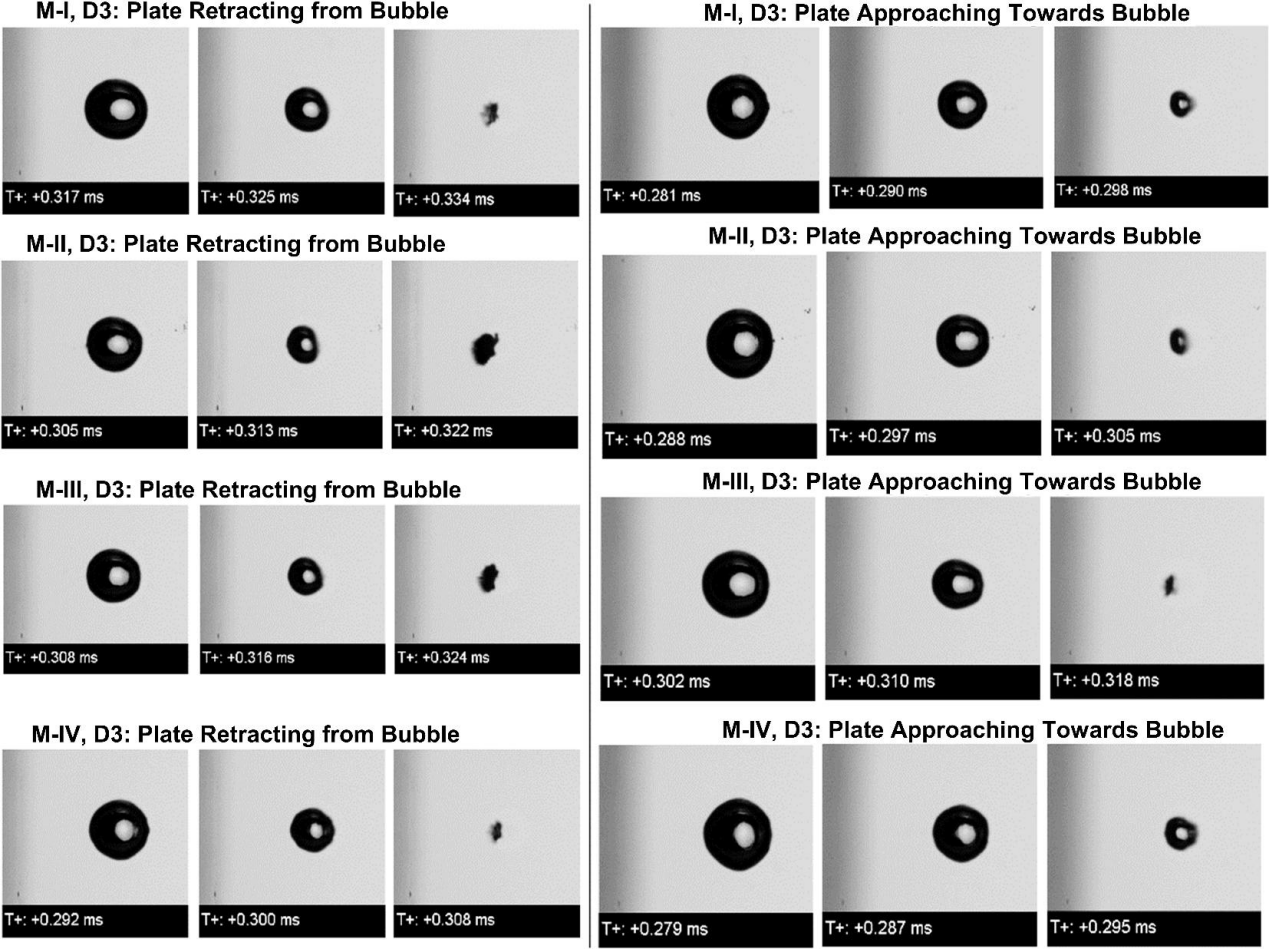
Comparison among the bubble shapes observed during four various modes of oscillation of plate in combination with the direction of plate motion for the distance between bubble center and plate equal to 1 mm.

The images for the mode I for the plate approaching towards collapsing bubble reflected an internal tiny conical structure, this indicates that the jet was initiated at a quiet centralized location and without affecting the peripheral surface thereby not showing a visual appearance of a notch. Careful observation revealed that a wider jet exists bridging the bubble and the plate surface in all cases if the bubble collapses near a moving plate irrespective of the direction of motion. For the plate approaching towards collapsing bubble, mode I and II showed thick distinct jets between the bubble and wall impacting on the solid boundary. However prior notch formation was not observed before collapse. With a higher mode number, the amplitude of oscillation also increases, thereby with increasing higher oscillation amplitude, the widening of the jet is observed for both directions of oscillations. However, modes III and IV did show clear jets but in earlier cases and it seems that the jetting occurs quite earlier before collapse (see an enlarged view of the jet in images) and attached to a plate almost in all cases. In both directions of plate cases and all reflected the plate-attached flattened cavities representing toroidal collapse. Overall, comparisons and careful observation identified the jetting on the surface and widening effects by increasing the oscillation amplitude of near plate bubbles. In the next case of D3 that is initial distance between the moving plate and bubble was 3 mm, Fig. 5 summarizes the imaging sequences just before collapsing for all modes of oscillation and both direction motion of the plate. In general, in some cases the bubble attained an oval shape before collapsing and the collapse looks like a concentric collapse.

The concentric collapse significantly radiates out the shock wave because of a stronger collapsing event. The post-collapse images in Fig. 2 (for D3) indicate that even though the collapse seems concentric, weaker jetting had occurred. This is reflected by the appearance of a tiny counter jet and conical extrusion of the bubble surface towards the oscillating plate, however second rebound and collapse were not in scope. Overall, one can hardly figure out the effects of the bubble at a distance of 3 mm (relative wall distance of 2) because no clear notch formation took place before the collapse and the possibility of stronger jetting on the surface is questionable. The conclusion drawn from the investigated case for larger relative wall distance is that the jetting on the wall. However, the visual appearance presented in all the above images from Figs. 1, 2, 3, 4 and 5 are of interest while excursing the manipulation methods for bubble dynamics. The clear differences are highlighted in imaging sequences qualitatively. For more detailed information, the quantitative analysis of collapse in all cases is necessary. The manipulation of bubble dynamics in the scope of quantitative analysis is referred to as either speeding up the bubble dynamics to get more efficient destructive effects or slowing down the bubble dynamics to minimize and delay the destructive effects.

**Fig. 6:**
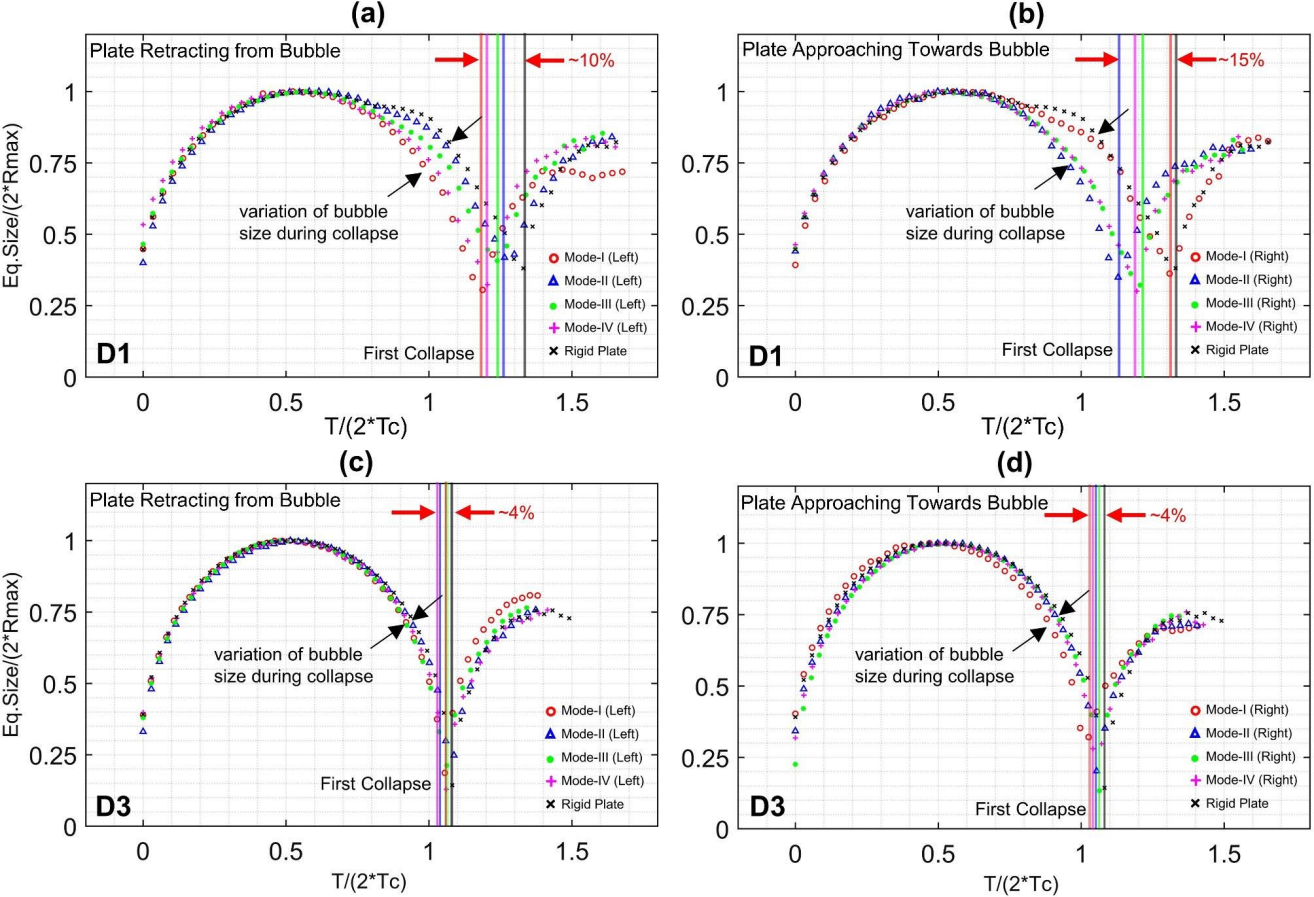
Comparison of normalized collapsing time for various cases of oscillation modes and plate’s motion direction in case of two different initial distances between the bubble and the plate.

The speed of collapse can be correlated to the strength of collapse. Therefore, we compared the normalized collapse time of the bubble in each case for the plate approaching towards and retracting from bubble with the collapse times of the bubble near the stationary plate. The comparison clearly indicates that the bubble’s collapse can be sped up by oscillating the nearby plate. The lesser the initial distance between the bubble and the plate, the more effective is the manipulation of the speed of bubble collapse. For larger initial distances between the bubble and the plate, the speed of the bubble can be still manipulated up to a certain extent. The comparison in Fig. 6 showed that in our demonstrated test cases bubble’s collapse was sped up by 4–15%. For a lesser distance where the bubble collapse can be effectively speeded up, the plate approaching towards bubble was a more significant effect that the plate retracting from bubble.

If we look at the effects of the amplitude of plate oscillation on collapse times, the modes with lesser amplitudes (M-I) were more effective and when the plate was retracting; while the higher amplitude was less effective. This signifies that the plate retracting from collapsing bubble with higher amplitude increases the effective relative wall distance for instance, still, the bubble’s collapsing time was not reduced. On the other hand, the lesser amplitude of the moving plate does not change the relative wall distance significantly still the bubble collapse time was reduced. This finding is contradictory to the relation between relative wall distance and collapse times. Because it can be seen that for D1 i.e. lesser relative wall distance the collapsing time is higher compared to the D3 case where relative wall distance is higher resulting in shorter collapsing time.

Overall, the bubble’s collapse near the plate surface at a distance of 1 mm can be speeded up by ~ 15% and at a 3 mm distance up to ~ 4%. The smaller the amplitude of oscillation, the bubble collapse can be accelerated by producing a bubble near the oscillating plate retracting from the collapsing bubble. And for higher amplitudes of plate oscillation, the bubble’s dynamics can be accelerated by generating a bubble near plates approaching towards bubble. However, this relationship is consistent expect some discrepancies, especially for lower relative wall distance. The major reason for accelerated collapse is the flow field generated by oscillating plate. The oscillatory plate resulted into higher pressure gradient (than the bubble collapsing near stationary plate) along the collapsing axes of bubble. We believe that further validation, extension and numerical simulation of our study will be breakthrough research for manipulating bubble dynamics. In general, our article opens up a new way of manipulating the bubble dynamics, specifically obtaining faster collapses of the bubble to achieve efficiency in applications such as microinjection or actuation. Our study also postulates that the cavitation near oscillating boundaries such as loaded oscillating propellers and impellers, destructive effects can be highly expected. For further clarity, the authors propose an in-depth future study to find a correlation between amplitude and oscillation frequencies of the plates based on current data and thereby expected manipulation effects on bubble dynamics. The results can also be used to further validate numerical method as benchmark.

## Methods

**Fig. 7:**
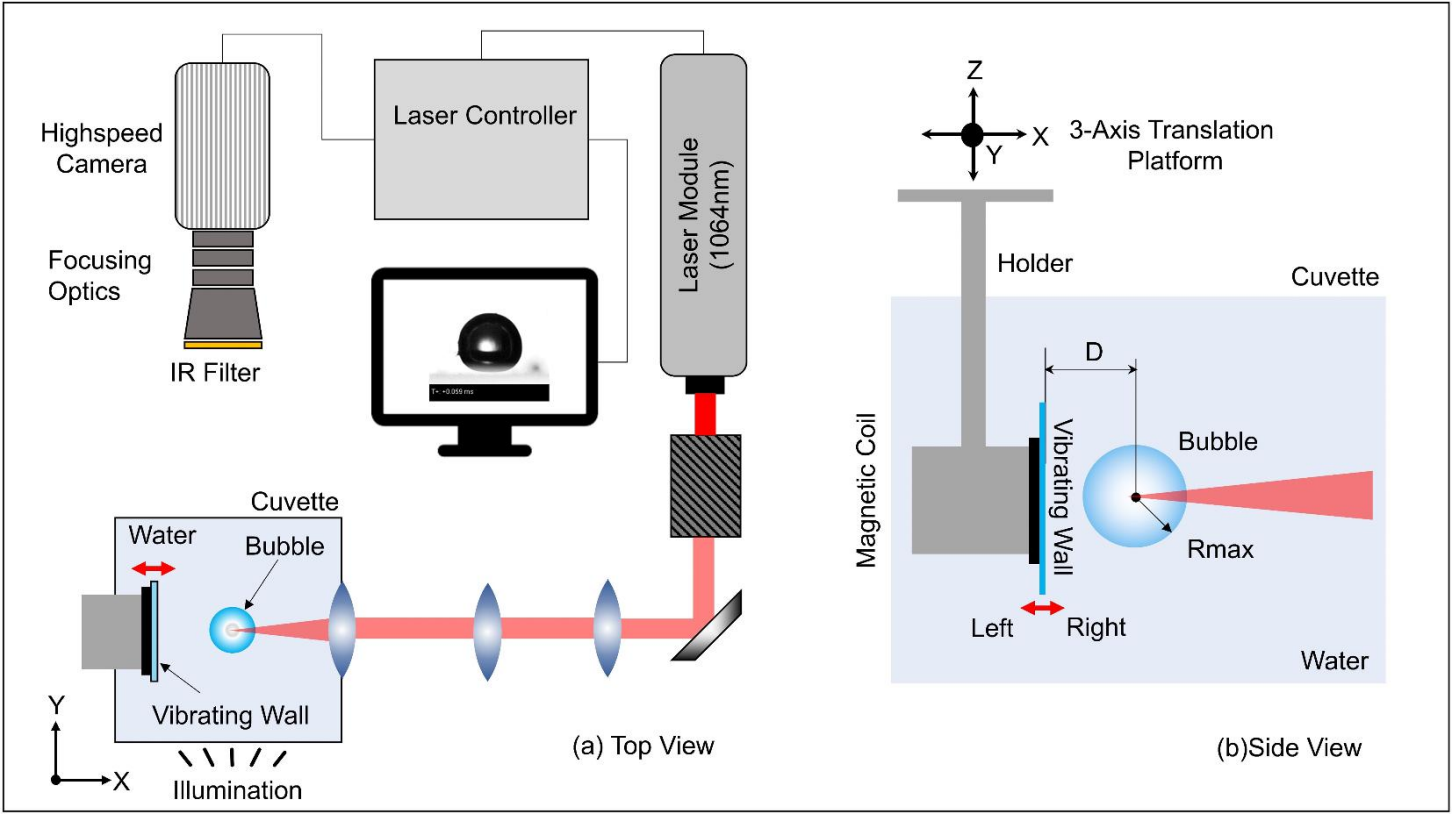
Illustration of experimental setup including laser, focusing lenses, cuvette, imaging system, and plate assembly.

In this work, we performed an experimental investigation of bubble dynamics near the oscillation wall. The detailed experimental setup is presented schematically in Fig. 7. Each test case has been repeated at least 5 times during tests to check the similarity of bubble dynamics in the results. A single bubble was generated inside the cuvette with dimensions of 50X50X50mm^3^, which was filled with distilled water. The laser beam of a Q-switched Nd:YAG pulsed laser with a pulse width of 10 ns and wavelength of 1064 nm was focused at a point by the lens system. The lens system was based on multiple converging and diverging lenses to avoid optical aberration and increase the focusing angle of a laser beam. This facilitated the generation of single confined plasma and generated a single distinct cavitation bubble. An aspherical focusing lens embedded on the wall of the cuvette receives the laser beam from the optical lens system and focuses the laser beam further into the water to generate plasma. The average pulse energy was about 22 ± 2 mJ. An optical-grade flat glass plate was placed vertically inside the cuvette. The glass plate was attached with a magnetostriction coil having variable oscillating frequencies and amplitudes. The assembly of the magnetic coil and plate was attached to the three axes’ linear translation platform to achieve the desired location in the cuvette using a rod. The surface of the plate had a distance ‘d’ to the bubble center, i.e. location of plasma seeding and we varied it to 1 mm and 3 mm in our test cases.

A digital single-lens reflex camera positioned to the 45° mirror to capture the bottom by the reflection. In this way, we located the position of the cavitation bubble with respect to the lateral position of the plate surface. The glass plate was aligned to the bubble center at the middle position of the glass plate using a three-axis traverse. The ratio between the size of the optical glass and the diameter of the cavitation bubble was large enough (approx. 8 times) to ignore the eddy vortex effect on the bubble dynamics. To capture ultrafast bubble dynamics, a high-speed camera Phantom V2012 equipped with focusing optical lenses and an IR filter aligned with the plasma and plate. The bubble dynamics was captured with a framing rate of 120 kHz (*Δt* = 8.33 µs) and an exposure time of 2 µs. The camera was initially focused on the plasma-located plane and the resolution of the camera was calibrated using a precise printed calibration grid on transparent glass. Our images resolve about 20 px/mm, and we kept it constant all over the test campaign. A LED illumination source in combination with a sand-polished glass diffuser served as background illumination. The diffused light shadowed the bubble through a transparent plate and water towards the camera sensor generating a shadowgraphic image of the bubble. For the first set of experiments, the bubble dynamics was captured near a stationary plate where the magnetostriction coil was not in action. In further test cases, the optical glass was forced to oscillate with defined frequencies and amplitude, and bubble dynamics along with the displacement of the plate was synchronously measured. Table 1 presents 4 different modes of the displacements of the oscillation wall with frequency and amplitude.

The location of plasma was considered as the origin of the cavitation bubble. The distance between the cavitation bubble and the plate (d) was defined by the reading of the distance between the plasma and the initial optical wall surface before the forced oscillation movement. The amplitude of oscillation was about 1–4% of the bubble diameter. In addition, we calculated the relative wall distance gamma (γ) by the ratio between the initial plate distance (distance between bubble center and plate surface) and the maximal bubble radius.

**Fig. 8:**
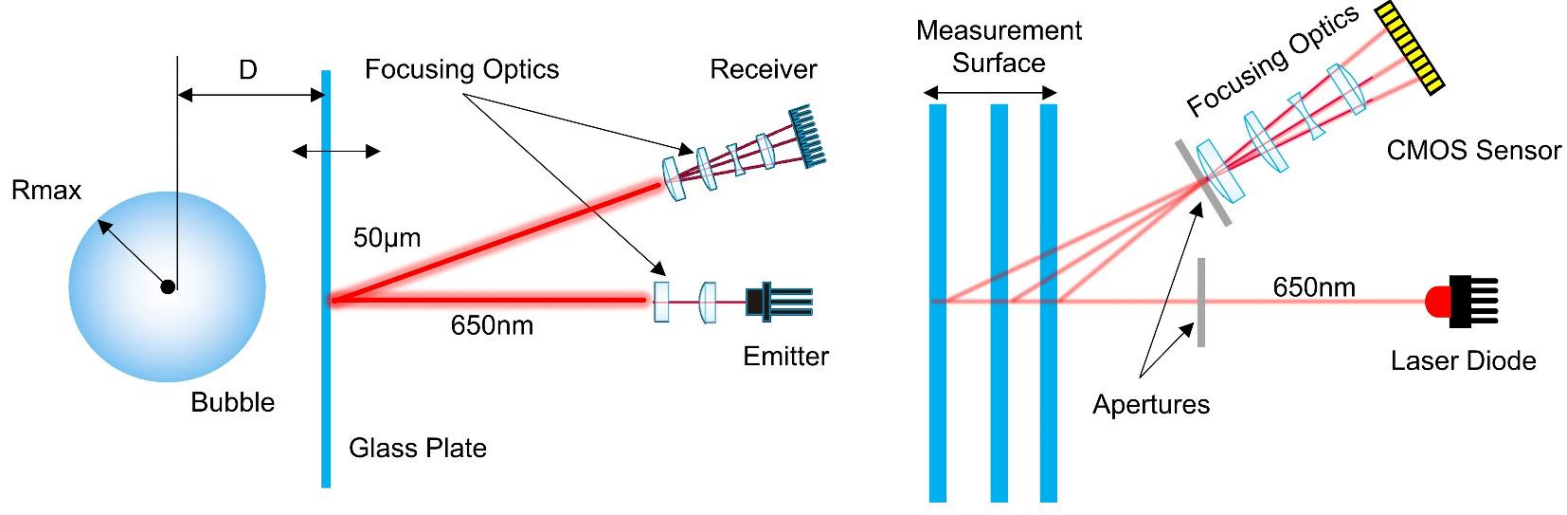
Measurement Principle of an optical displacement measurement device.

To measure the oscillating frequency and amplitude, we relied on the optical highspeed displacement sensor. The working principle of the sensor is shown in Fig. 8. The displacement sensor was based on the optical ray intensity measurement technique. The reflected light from the target plate was entered through the apertures of the sensor and then focused on the linear CMOS sensor. By detecting the location of the high-intensity point on the sensor, the position of the target plate was then determined. The displacement sensor was synchronized with the laser pulse therefore we were able to accurately able to determine the direction of motion of the plate when the laser was seeded into water. In addition, the careful observation of high images also revealed the direction of the motion plate for additional confirmation.

The cavitation bubble was recorded by the high-speed camera and analyzed with an image processing program. The spatial parameters of the cavitation bubble and the maximal radius of the cavitation bubble were determined by image processing and previously estimated calibration. The fluctuation in the laser beam, local properties of water, and impurities in water might have introduced the variation in the size of the bubble, however, it was limited to ~ 3% in our experiments. We compared our quantitive results such as bubble’s equivalent size vs collapse time in non-dimensional form. The growing bubble did not remain spherical as it approached the aluminum foil. Therefore, we measured its equivalent size based on its longitudinal and lateral radial sizes. Here the equivalent size of bubble is the average of longitudinal and lateral radial sizes of bubble. The bubble’s equivalent size was non-dimensionalize with maximum equivalent radial size and collapsing time with Rayleigh collapse times (${{T}_{c}}^{Rayleich}$). In Rayleigh’s derivation of bubble collapse inside fluid with density $\rho$ at ambient pressure converted to the kinematical energy within a converging flow lied. As the bubble reached its maximum radius ${R}_{max}$, it started to collapse at the differential pressure $\Delta p=({p}_{a}-{p}_{i})$. The ${p}_{a}$ is the ambient pressure and the ${p}_{i}$ is the interior pressure of the cavitation bubble. The interior pressure ${p}_{i}$ of cavitation bubble at maximum expansion could be considered as the saturation pressure ${p}_{v}$ (Reuter 2022). We could express the Rayleigh collapse time of bubble ${{T}_{c}}^{Rayleich}$ as a followed equation:

$${{T}_{c}}^{Rayleich}=0.91468 {R}_{max} \sqrt{\rho /({p}_{a}-{p}_{v})}$$

The test matrix as shown in the following table summarizes the test we performed in our investigations. Each test case was repeated at least 5 times. The radial size of bubble varies 0.1 mm (~ 3%) and the vibrating plate motion have error of 3—4.5 µm (3–7%).

## Data Availability

All data generated or analyzed during this study are included in this article, other additional datasets used during the current study is available from the corresponding author on reasonable request.
